# The Hidden Players of the Fecal Metabolome: Metabolic Dysregulation Beyond SCFAs Under a High-Fat Diet

**DOI:** 10.3390/metabo15100660

**Published:** 2025-10-07

**Authors:** María Martín-Grau, Pilar Casanova, José Manuel Morales, Vannina González Marrachelli, Daniel Monleón

**Affiliations:** 1 Departament de Patologia, Universitat de València, 46010 Valencia, Spain; pilar.casanova@uv.es (P.C.); j.manuel.morales@uv.es (J.M.M.); 2 INCLIVA Biomedical Research Institute, 46010 Valencia, Spain; 3 Departament de Fisiologia, Universitat de València, 46010 Valencia, Spain; vannina.gonzalez@uv.es

**Keywords:** metabolomics, MASLD, fecal metabolites, host–microbiota interactions, NMR

## Abstract

**Background/Objectives:** The interplay between host metabolism and gut microbiota is central to the pathophysiology of metabolic diseases, including metabolic dysfunction-associated steatotic liver disease (MASLD). In this study, we investigated the underexplored fecal host–microbiota co-metabolism profile of male and female Wistar rats after 21 weeks of high-fat diet (HFD), a model previously validated for early MASLD. **Methods:** Using ^1^H-NMR spectroscopy, we detected and quantified metabolites in fecal samples associated with hepatic metabolism beyond short-chain fatty acids (SCFAs), such as energy-related metabolites, amino acid turnover, branched-chain amino acid (BCAA) catabolism, and microbial fermentation. **Results:** Distinct metabolic signatures were identified according to diet and sex, and statistical analysis was performed. Notably, alterations were observed in bile acids (BAs) such as cholate and glycocholate, suggesting disruptions in enterohepatic circulation. The presence of fucose, a sugar linked to liver pathology, was also elevated. Energy-related metabolites indicated a shift from lactate production to increased acetoacetate and malonate levels, implying redirection of pyruvate metabolism and inhibition of the TCA cycle. BCAA derivatives such as 3-methyl-2-oxovalerate and 3-aminoisobutyrate were altered, supporting earlier findings on disrupted amino acid metabolism under HFD conditions. Furthermore, microbial metabolites including methanol and ethanol showed group-specific differences, suggesting shifts in microbial activity. **Conclusions:** These findings complement previous longitudinal data and provide a functional interpretation of newly identified metabolites. These metabolites, previously unreported, are now functionally contextualized and linked to hepatic and microbial dysregulation, offering novel biological insights into early MASLD mechanisms.

## 1. Introduction

Hepatic steatosis, or the accumulation of fat in the liver, is a hallmark feature of metabolic-associated fatty liver disease (MASLD) progression [1], which has become a prevalent condition worldwide [2]. In recent years, with the redefinition of the term [3], MASLD has come to be understood as a metabolic disease. One of the most widely studied aspects in this context is the alteration of cellular metabolism, often explored through metabolomic approaches. The presence of metabolites in various biological samples (such as blood, urine, or feces) can serve as a reflection of changes in hepatic metabolism [4].

It has been widely demonstrated that dietary alterations, in the context of MASLD, lead to significant changes in the gut microbiota and its metabolism, which play a key role in driving or modulating hepatic metabolic dysfunction [5]. For this reason, the analysis of fecal samples remain highly attractive in this field, as it can provide valuable insights into host–microbiota interactions. Research has mainly focused on the role of high-abundance metabolites associated with liver disease such as short-chain fatty acids (SCFAs), but also branched-chain amino acids (BCAA), choline, or trimethylamine N-oxide (TMAO) [6]. Nonetheless, numerous other metabolites may also hold significant relevance in the investigation of MASLD, although they remain underexplored. This study focuses on uncharacterized fecal metabolites and their biological significance, especially in relation to hepatic and microbial function. Finally, to gain a more integrative understanding of how these novel metabolites relate to microbial function, correlation analyses were performed using previously published microbiota data [7].

## 2. Materials and Methods

### 2.1. Animal Model and Housing

**Figure 1:**
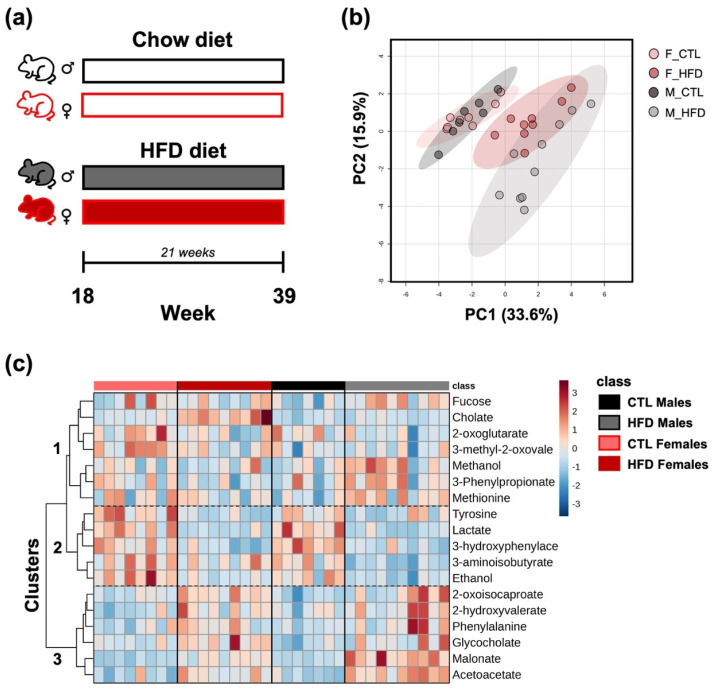
Multivariate and univariate analysis of fecal metabolite profiles across experimental groups. (**a**) In vivo model representation (n/group = 7–10 animals/per group). (**b**) PCA based on fecal metabolite profiles after 21 weeks on HFD. PC1 and PC2 explain 33.6% and 15.9% of the total variance, respectively. (**c**) Heatmap of the fecal metabolite profiles across groups, selected based on ANOVA results. Rows correspond to individual metabolites, and columns represent individual samples. Color intensity indicates relative abundance, with red indicating higher and blue indicating lower levels. Abbreviations: F_CTL, CTL females; F_HFD, HFD females; M_CTL, CTL males; M_HFD, HFD males; 3-hydroxyphenylace, 3-hydroxyphenylacetate; 3-methyl-2-oxovale, 3-methyl-2-oxovalerate.

Animal handling and dietary interventions were conducted as previously described [7]. Also, parameters of disease progression were previously measured and reported. These measurements validate the MASLD model used in the present study [7]. Briefly, seventeen-week-old male and female Wistar rats (Janvier Labs, Le Genest-Saint-Isle, France) were acclimatized for one week and then randomly assigned to control (CTL) or high-fat diet (HFD) groups for 21 weeks (n/group = 7–10 animals/per group) (Figure 1a). For the present study, only fecal samples collected at the endpoint (week 21) were analyzed. All animal procedures were approved by the Ethics Committee for Experimental Research of the University of Valencia, Spain (approval code: 2019/VSC/PEA/0129-A1538561308126/type2, date of approval: 19 June 2019).

### 2.2. Metabolomics by Proton (^1^H) Nuclear Magnetic Resonance (NMR) and Metabolite Interpretation

**Figure 2:**
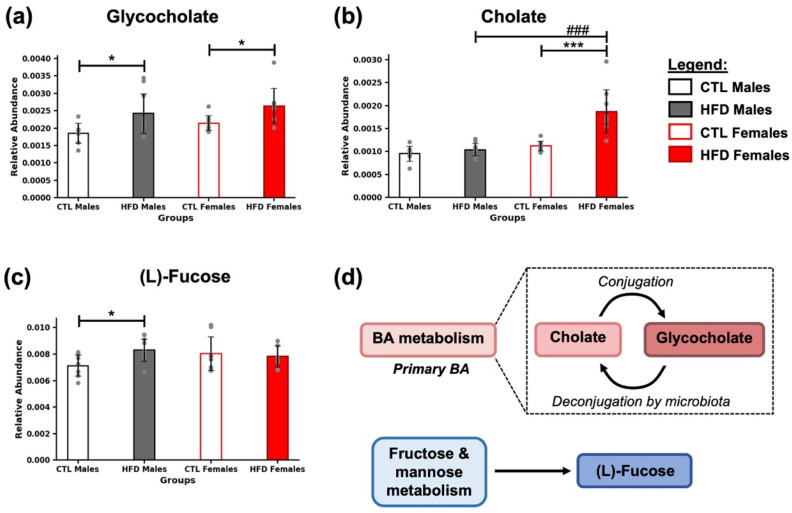
Alterations in bile acid and fucose-related metabolites after 21 weeks of HFD. Bar plots show the relative abundance of (**a**) glycocholate, (**b**) cholate, and (**c**) (L)-fucose across experimental groups (CTL males, HFD males, CTL females, HFD females) at week 21. Data are presented as mean ± SD. Two-way ANOVA and Bonferroni post hoc test were used in (**b**,**c**), while Kruskal–Wallis test followed by Mann–Whitney U test was used for (**a**). Statistical significance was set at * *p* < 0.05, *** *p* < 0.001 (CTL vs. HFD), and ### *p* < 0.001 (male vs. female). (**d**) Metabolites were grouped according to their main associated metabolic pathways. Glycocholate and cholate are involved in bile acid (BA) metabolism, whereas (L)-fucose is associated with fructose and mannose metabolism.

**Figure 3:**
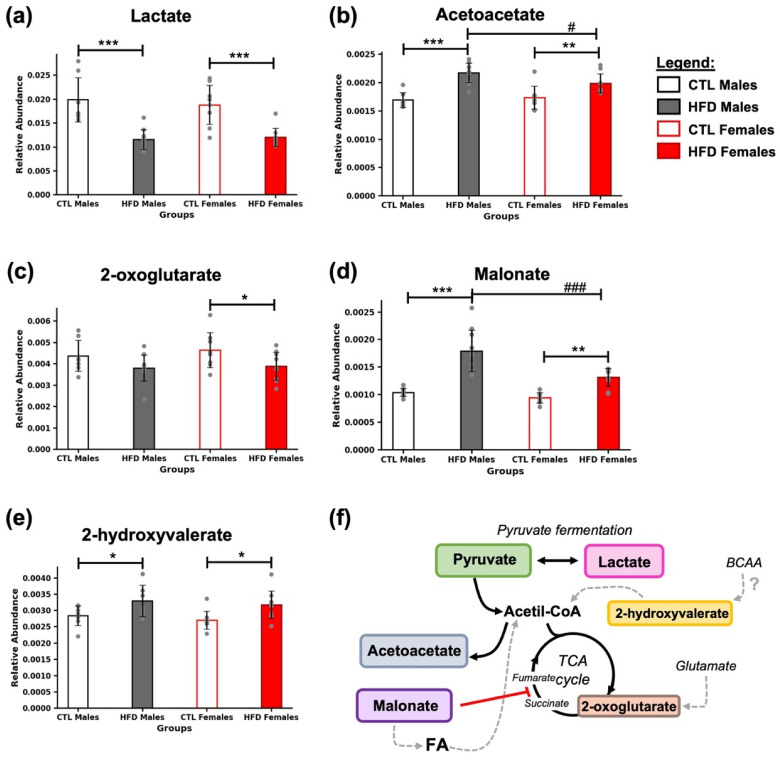
Energy-related metabolic alterations induced by 21 weeks of HFD. Bar plots represent the relative abundance of (**a**) lactate, (**b**) acetoacetate, (**c**) 2-oxoglutarate, (**d**) malonate, and (**e**) 2-hydroxyvalerate in fecal samples across experimental groups. Data are expressed as mean ± SD. Statistical significance (based on two-way ANOVA and Bonferroni post hoc test) was set at * *p* < 0.05, ** *p* < 0.01, and *** *p* < 0.001 (CTL vs. HFD) and # *p* < 0.05, and ### *p* < 0.001 (male vs. female). (**f**) Metabolites were grouped based on their role in energy metabolism. Black arrows indicate the direction of the reactions, gray dashed arrows represent hypothetical reactions that may occur, the red line denotes an inhibitory reaction, and the question mark indicates an uncertain origin from the BCAAs. Abbreviations: BCAA, branched-chain amino acids; FA, fatty acids; TCA cycle, tricarboxylic acid cycle.

**Figure 4:**
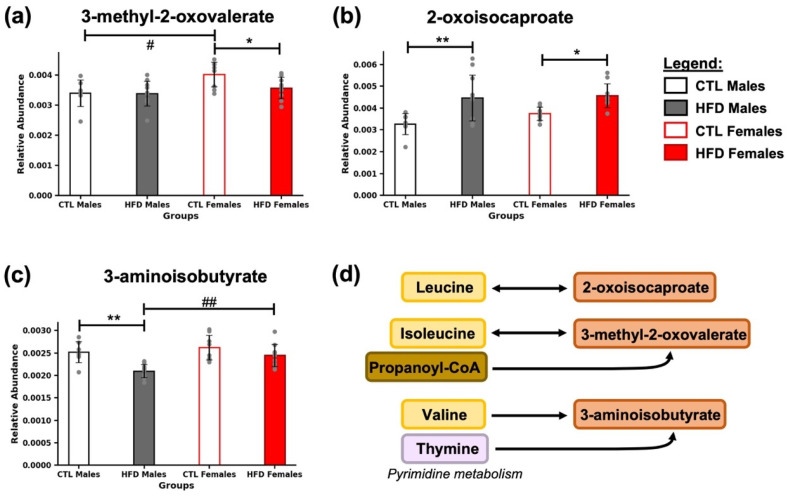
Alterations in intermediates of BCAA metabolism after 21 weeks of HFD. Bar plots represent the relative abundance of (**a**) 3-methyl-2-oxovalerate, (**b**) 2-oxoisocaproate, and (**c**) 3-aminoisobutyrate across experimental groups (CTL males, HFD males, CTL females, HFD females) at week 21. Data are expressed as mean ± SD. Statistical significance (based on two-way ANOVA and Bonferroni post hoc test) was set at * *p* < 0.05, ** *p* < 0.01 (CTL vs. HFD) and # *p* < 0.05, ## *p* < 0.01 (male vs. female). (**d**) Metabolites were grouped according to their association with key pathways related to BCAA (leucine, isoleucine, valine) catabolism and minor intermediates.

**Figure 5:**
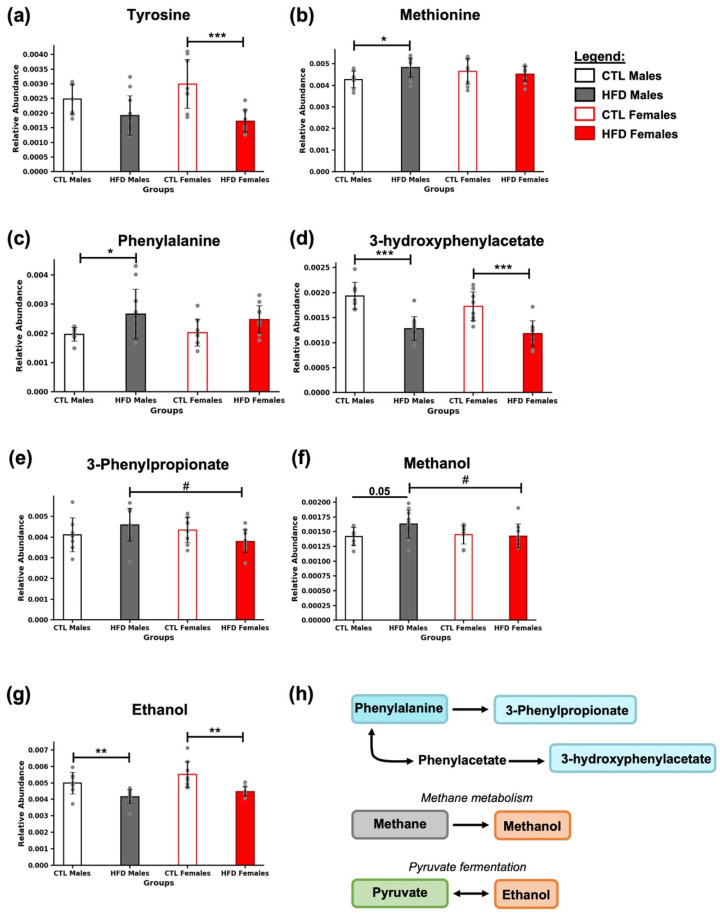
Amino acid profiles and microbial-derived metabolites in response to HFD. Bar plots represent the relative abundance of (**a**) tyrosine, (**b**) methionine, (**c**) phenylalanine, (**d**) 3-hydroxyphenylacetate, (**e**) 3-phenylpropionate, (**f**) methanol, and (**g**) ethanol in fecal samples across experimental groups. Data are expressed as mean ± SD. Statistical significance (based on two-way ANOVA and Bonferroni post hoc test) was set at * *p* < 0.05, ** *p* < 0.01, and *** *p* < 0.001 (CTL vs. HFD) and # *p* < 0.05 (male vs. female). The *p*-value for methanol (**f**) between CTL and HFD males was 0.05, as indicated in the figure, although it did not reach the significance threshold. (**h**) Metabolites are color-coded according to their metabolic pathways: phenylalanine-derived metabolites (blue), metabolites involved in methane metabolism (gray), and those related to microbial fermentation from pyruvate (green). Alcohols are marked in orange.

**Figure 6:**
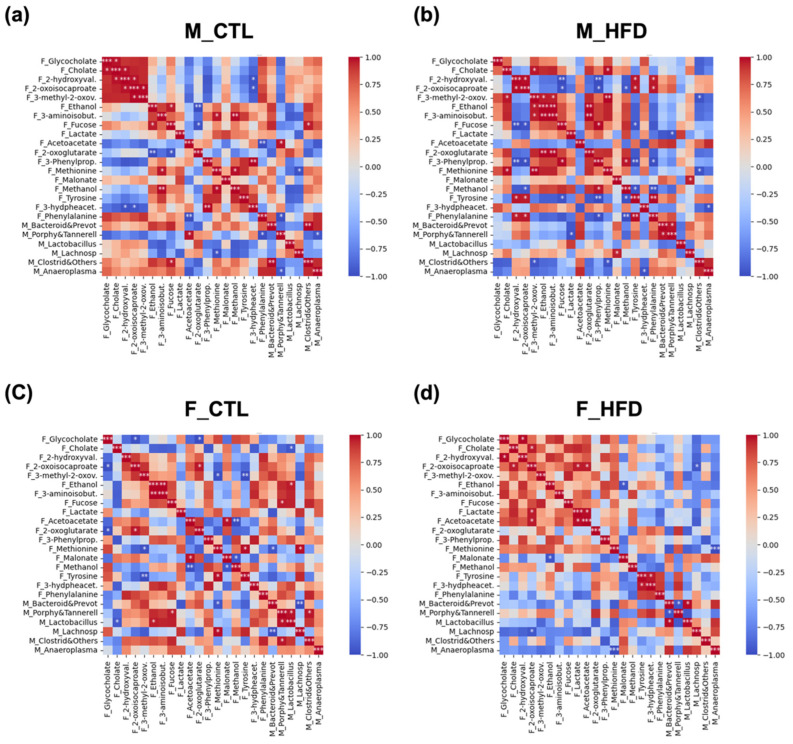
Correlation matrices of fecal metabolites and microbial taxa across experimental groups after 21 weeks of HFD. Color-scaled correlation matrices were generated separately for each experimental group to visualize associations between measured fecal metabolites and microbial taxa: (**a**) CTL males (M_CTL), (**b**) HFD males (M_HFD), (**c**) control females (F_CTL), and (**d**) HFD females (F_HFD). Pearson correlation coefficients are represented by the color intensity, ranging from negative (blue) to positive (red), highlighting group-specific metabolite–microbe association patterns. Statistical significance is indicated by asterisks within cells: *p* < 0.05 (*), *p* < 0.01 (**), *p* < 0.001 (***). The main diagonal (*r* = 1.0) corresponds to self-correlations and was excluded from interpretation. Abbreviations: F_, fecal metabolites data; F_2-hydroxyval., 2-hydroxyvalerate; F_3-hydpheacet., 3-hydroxyphenylacetate; F_3-methyl-2-oxov., 3-methyl-2-oxovalerate; F_3-aminoisobut., 3-aminoisobutyrate; F_3-Phenylprop., 3-Phenylpropionate; M_, microbiota data; M_Anaeroplasma, *Anaeroplasma genus*; M_Bacteroid&Prevot, Bacteroidaceae and Prevotellaceae, M_Clostrid&Others, Clostridiaceae and others, M_Lactobacillus, *Lactobacillus genus*, M_Lachnosp, Lachnospiraceae family; M_Porphy&Tannerell, Porphyromonadaceae and Tannerellaceae.

All procedures related to ^1^H-NMR measurements and data analysis were carried out following the same methodology previously described in Martin-Grau, M et al. 2025 [7]. In the present study, only underexplored metabolites present in fecal samples collected after 21 weeks were considered. Briefly, feces underwent a prior aqueous extraction process. Specifically, 50–60 mg of feces was mixed with 100 µL of distilled water. After several freeze/thaw cycles and multiple centrifugations at 14.800 rpm for 5 min, the supernatant was collected and stored at −80 °C until use. Next, 20 µL of the fecal supernatant was mixed with 4 µL of phosphate buffer (1.5 M of potassium dihydrogen phosphate (KH_2_PO_4_) (795488, Sigma-Aldrich, Darmstadt, Germany), and 5.8 mM of 3-(trimethylsilyl)-2,2,3,3-tetradeutero-propionic acid (TSP) (11202, Deutero, Kastellaun, Germany) in sterile deuterium oxide (D_2_O) (1.13366, Sigma, St. Louis, MO, USA; pH 7.4), and only 20 µL of this mixture was measured by a 600.13 MHz Bruker AVANCE III NMR spectrometer (Bruker BioSpin GmbH, Rheinstetten, Germany) using the 1 mm triple resonance (TXI) probe. After acquisition, spectra were first preprocessed using MestReNova software (v14.1.1, Mestrelab Research S.L, Santiago de Compostela, Spain). Here, we corrected the spectra based on the phase, baseline, and reference to the second peak of the alanine (1.478 ppm). Metabolite assignment to each peak of the spectra was performed with Chenomx NMR Suite (v8.1, Chenomx Inc., Edmonton, AB, Canada) and confirmed by consulting the main databases: the Human Metabolome Database (HMDB) and the Kyoto Encyclopedia of Genes and Genomes (KEGG). The underexplored metabolites selected for this study are listed in Table 1, along with their corresponding annotations in HMDB and KEGG. As can be observed, the same metabolite may display several peaks throughout the ^1^H-NMR spectrum depending on the number of hydrogens and their chemical environment (with the exception of those considered singlets). Among the possible peaks for each metabolite, a reference peak was selected (highlighted in bold in Table 1) based on minimal or no overlap with other signals in the spectrum. The area under each reference peak was calculated using MATLAB software (MATLAB R2014a, MathWorks, Natick, MA, USA) with in-house scripts, and the resulting values were used for statistical analyses and figure generation. The data were not subjected to further normalization, as all samples were diluted with the same volume of water during the extraction. The data are available in the Supplementary Materials Section. All metabolic pathways represented in the figures (Figure 2, Figure 3, Figure 4, Figure 5 and Figure 6) were cross-referenced using HMDB, KEGG, and references [5]. These pathways should be understood as an interpretation of the data.

### 2.3. Microbiota Analysis

In addition, an integrative correlation analysis was performed using microbiota data previously published from the same animal cohort at week 21 [7]. Briefly, bacterial genomic DNA was extracted from fecal samples using the commercial kit QIAamp Fast DNA Stool Mini Kit (51604, QIAGEN N.V., Hilden, Germany). Primers were optimized for the bacterial families of interest (*Anaeroplasma genus*, Bacteroidaceae and Prevotellaceae families, Clostridiaceae and other families, *Lactobacillus genus*, Lachnospiraceae family, Porphyromonadaceae and Tannerellaceae families), and quantitative PCR (qPCR) analyses were performed to determine their relative abundances based on Ct, ∆Ct, ∆∆Ct, and 2^−∆∆Ct^ values. Full details of the methodology can be found in the previously published article. The sample size per group was 4 animals, and the data used to establish the correlation tables are available in the Supplementary Materials Section of this article.

### 2.4. Statistical Analysis

Statistical analysis was performed using SPSS Statistics version 29 (IBM SPSS Statistics 29.0, New York, NY, USA) and as described previously [7]. Briefly, the Kolmogorov–Smirnov test was applied to assess the normality of data distribution. For metabolites showing a normal distribution, two-way ANOVA was conducted with Bonferroni post hoc corrections to evaluate the effects of diet (CTL vs. HFD) and sex (males vs. females). Glycocholate was the only metabolite that did not follow a normal distribution. In this case, non-parametric tests for independent samples were applied: first, the Kruskal–Wallis test was used to assess overall group differences, followed by the Mann–Whitney U test for pairwise comparisons. In all cases, differences were considered statistically significant at a *p*-value under 0.05. Diet-dependent differences were indicated with asterisks (*), while sex-related differences were marked with a hash symbol (#). Principal Component Analysis (PCA) and heatmap generation were performed using MetaboAnalyst 6.0 (accessed on 20 June 2025: https://www.metaboanalyst.ca/). Prior to analysis, data were auto-scaled. For PCA, principal components 1 and 2 (PC1 and PC2) were used. The heatmap was generated based on the ANOVA results [7]. Correlation matrices were generated using BioPython version 1.85 with pandas for data handling, scipy.stats (*pearsonr*) for computing Pearson’s correlation coefficients and *p*-values, and seaborn and matplotlib for visualization. Pearson correlation coefficients were calculated for each experimental group to evaluate the associations between metabolites and microbial taxa within the same condition. The resulting correlation matrices were visualized as color-scaled heatmaps. This analysis aimed to explore potential biological associations between microbial composition and the novel metabolic alterations described in this paper.

## 3. Results

### 3.1. Group Segregation by Fecal Underexplored Metabolite Profiles After 21 Weeks of HFD

PCA revealed a clear separation of the four experimental groups based on their fecal metabolite profiles, particularly along PC1 (33.6% of the variance) and PC2 (15.9%) (Figure 1b). Notably, samples from the CTL groups clustered separately (left) from those of the HFD (right), indicating a marked shift in the metabolic composition after 21 weeks. Furthermore, HFD males and females segregate from each other. The heatmap displays the relative abundance of selected metabolites that were significantly different across groups based on ANOVA after 21 weeks on HFD (Figure 1c). Metabolites were grouped into three major clusters, with clusters 2 and 3 exhibiting a clearer pattern of relative concentration differences between groups. Metabolites in cluster 2 appear to be elevated in the CTL groups, whereas metabolites in cluster 3 seem to be increased in the HFD groups. However, cluster 1 appears to display more sex-dependent patterns. For example, methanol, methionine, and 3-phenylpropionate seem to be elevated in HFD males but not in HFD females. Similarly, 2-oxoglutarate and 3-methyl-2-oxovalerate appear to be present at higher concentrations in CTL females while showing lower concentrations in CTL males.

### 3.2. Endogenous Metabolites Derived from Hepatic Metabolism

After 21 weeks of HFD, significant alterations were observed in the relative abundance of several metabolites associated with hepatic bile acid (BA) and carbohydrate metabolism (Figure 2). Glycocholate and cholate, two key metabolites involved in BA pathways (Figure 2d), showed increased levels in HFD groups (* *p* < 0.05, Figure 2a; *** *p* < 0.001, Figure 2b), with the most pronounced effect observed in HFD females (### *p* <0.001) (Figure 2b). Additionally, L-fucose, a sugar related to carbohydrate metabolism (Figure 2d), was significantly increased in HFD males compared to CTL males (Figure 2c). Together, these results suggest that a HFD for 21 weeks induces specific changes in metabolites linked to hepatic metabolism, highlighting the interplay between host and microbiota.

### 3.3. Metabolites Related to Energy Metabolism

In fecal samples collected after 21 weeks of HFD, several metabolites related to energy metabolism were detected (Figure 3). All of them are directly or indirectly connected to the pyruvate–acetyl-CoA axis and the tricarboxylic acid (TCA) cycle (Figure 3f). A marked decrease in lactate levels was observed in HFD animals (*** *p* < 0.001) (Figure 3a), along with an increase in acetoacetate in both HFD males (*** *p* < 0.001) and females (** *p* < 0.01) (Figure 3b). A reduction in 2-oxoglutarate was detected in HFD females (* *p* < 0.05) (Figure 3c), while malonate levels increased significantly in both HFD males (*** *p* < 0.001) and females (** *p* < 0.01) (Figure 3d). Additionally, 2-hydroxyvalerate was elevated in both HFD males (* *p* < 0.05) and females (* *p* < 0.05) (Figure 3e). Moreover, differences were found between HFD males and HFD females in the levels of acetoacetate (# *p* < 0.05, Figure 3b) and malonate (### *p* < 0.001, Figure 3d).

### 3.4. Intermediates of BCAA Metabolism

At the end of the 21-week HFD exposure, several metabolites involved in BCAA metabolism were significantly altered (Figure 4), specifically, the abundance of 3-methyl-2-oxovalerate, 2-oxoisocaproate, and 3-aminoisobutyrate, intermediates in the catabolism of isoleucine, leucine, and valine, respectively (Figure 4c). 3-methyl-2-oxovalerate was significantly decreased in HFD females (* *p* < 0.05) (Figure 4a), 2-oxoisocaproate was significantly increased in HFD males (* *p* < 0.05) and HFD females (* *p* < 0.05) (Figure 4b), and 3-aminoisobutyrate was significantly decreased in HFD males (** *p* < 0.01) (Figure 4c), compared to their respective CTL groups. Additionally, sex-related differences were found in CTL animals for 3-methyl-2-oxovalerate (# *p* < 0.05) (Figure 4a), and in HFD animals for 3-aminoisobutyrate (## *p* < 0.01) (Figure 4c).

### 3.5. Presence of Amino Acids and Their Derivatives

In the samples, metabolites related to amino acid metabolism (methionine), aromatic amino acids (tyrosine and phenylalanine), and phenylalanine-derived compounds (3-hydroxyphenylacetate and 3-phenylpropionate) were identified (Figure 5). A decrease in tyrosine was observed in HFD females (*** *p* < 0.001) (Figure 5a), along with an increase in methionine and phenylalanine in HFD males (* *p* < 0.05) (Figure 5b,c). In contrast, 3-hydroxyphenylacetate levels were reduced in both HFD males and females (*** *p* < 0.001) (Figure 5d). For 3-phenylpropionate, only sex-related differences were found among HFD animals (# *p* < 0.05) (Figure 5e).

### 3.6. Presence of Characteristic Microbial Metabolites

Exposure to an HFD for 21 weeks significantly modulated the levels of microbial fermentation by-products in feces (Figure 5). Methanol levels were modestly elevated in HFD males compared to CTL males (*p* = 0.05). However, the *p*-value did not reach statistical significance. Differences were also observed between HFD males and females, with methanol levels being higher in males (# *p* < 0.05) (Figure 5f). Ethanol showed a significant decrease in HFD animals (** *p* < 0.01) (Figure 5g). These two compounds are specifically produced by the gut microbiota, either as derivatives of methanogenesis or as by-products of pyruvate fermentation (Figure 5h).

### 3.7. Correlation Analysis Among Newly Identified Metabolites and Microbiota

Correlation analysis revealed clear associations between newly identified fecal metabolites and microbial taxa previously characterized in the same animal cohort at week 21 [7] (Figure 6). In CTL males (Figure 6a), significant positive correlations were primarily observed between BA, energy-related metabolites, and BCAA intermediate metabolites (e.g., cholate, 2-hydroxyvalerate, 2-oxoisocaproate). Moreover, these metabolites were positively correlated with bacterial genera except for Porphyromonadaceae and Tannerellaceae. In males fed an HFD (Figure 6b), the HFD group displayed a greater number of significant correlations, with a shift toward both stronger positive and stronger negative associations compared to CTL males. Metabolites such as phenylalanine, tyrosine, methionine, 3-phenylpropionate, and methanol gained greater relevance. Moreover, a positive correlation was observed between low lactate levels and low levels of *Lactobacillus* spp. In CTL females (Figure 6c), significant correlations were fewer and more restricted. Significant correlations were observed between ethanol, fucose, or methionine and selected bacterial taxa. Compared to CTL males, CTL females did not appear to exhibit such a well-defined pattern. In females under HFD conditions (Figure 6d), we observed an even more polarized pattern, with correlations clustering predominantly as either positive or negative compared to CTL females. Notably, HFD females exhibited a large cluster of positive correlations involving BA, BCAA metabolism intermediates, or ethanol, fucose, lactate, and related metabolites.

## 4. Discussion

Although the present study is focused on underexplored fecal metabolites rather than canonical SCFAs, it is important to note that these data were derived from an established in vivo model of early MASLD [7]. Consequently, the identified metabolites and metabolite–microbiota correlations should be interpreted within the pathophysiological context of diet-induced MASLD. Our analysis revealed distinct sex-dependent and diet-induced alterations in the abundance of various microbial and host-derived metabolites. The altered fecal metabolites identified in this study are likely to represent an effect of the long-term HFD intervention, as they reflect metabolic adaptations involving both bacterial and host pathways. Notably, metabolites associated with endogenous host-derived metabolites, energy metabolism, amino acid turnover, BCAA derivatives, and microbial fermentation were significantly affected by HFD, underscoring the complex interplay between host–microbiota co-metabolism, dietary intake, and gut microbial activity. These findings contribute to a growing body of evidence highlighting how chronic exposure to an HFD reshapes the gut metabolic environment, potentially influencing systemic metabolic homeostasis and disease risk.

Starting with the endogenous metabolites, BAs and fucose were detected in feces. First, cholate and glycocholate are synthesized in the liver and secreted via bile. Glycocholate, a conjugated BA, can be deconjugated by the gut microbiota, and its altered levels in fecal samples may reflect modifications in hepatic metabolism and disruptions of the enterohepatic axis [5]. Similarly, (L)-fucose is a sugar commonly used in protein glycosylation by host cells, which can be also used by microbiota [8,9]. It is related to the fructose and mannose metabolism (shown in KEGG). Fucose has been previously reported in urine samples from individuals with liver pathologies, including cirrhosis and alcoholic liver disease [10]. Moreover, it has been reported that supplementation of an HFD with L-fucose reduced steatosis in a C57BL/6 mouse model [11]. Elevated fecal levels of fucose may be indicative of hepatic dysfunction.

With respect to energy metabolism, a significant decrease in lactate was observed in HFD animals, suggesting a shift in pyruvate utilization, potentially redirected toward acetyl-CoA production, a key player in MASLD pathophysiology [12]. Additionally, one of the bacterial species associated with lactate production, *Lactobacillus* spp. [5], was found to be decreased as early as week 3 of HFD exposure [7]. Here, we saw a positive correlation of having decreased levels of lactate and a decrease in *Lactobacillus* spp. in relation to HFD and MASLD. Excess acetyl-CoA may be diverted toward acetoacetate synthesis, while entry into the TCA cycle may be downregulated, as suggested by reduced levels of 2-oxoglutarate (Figure 3f). Furthermore, malonate levels were elevated in HFD animals. Malonate serves both as a precursor for FA synthesis and as a known competitive inhibitor of succinate dehydrogenase [5], reinforcing the notion of a dampened TCA cycle activity. In parallel, 2-hydroxyvalerate, a compound that could be classified as a BCAA-related metabolite and linked to the TCA cycle [13], was also increased in feces.

Regarding BCAA metabolism, underexplored metabolites such as 3-methyl-2-oxovalerate, 2-oxoisocaproate, and 3-aminoisobutyrate are derived from the catabolism of isoleucine, leucine, and valine, respectively [14]. Notably, they have been associated with acetyl-CoA and propionyl-CoA metabolism and, by extension, with the TCA cycle and energy metabolism [13]. 3-aminoisobutyrate, in turn, may also originate from thymine catabolism, thereby also linking it to pyrimidine metabolism (Figure 4d). BCAA and thymine were reported as longitudinally increased in the HFD exposure and early MASLD model [7], further supporting their relevance in chronic metabolic adaptation to diet.

Aromatic amino acids and their derivatives were also impacted. Compounds such as tyrosine, methionine, and phenylalanine can result from incomplete protein digestion, increased protein catabolism, or excessive dietary intake under HFD conditions. However, their presence in feces also reflect the influence of bacterial metabolism. Metabolites derived from phenylalanine, including 3-hydroxyphenylacetate and 3-phenylpropionate, further support a microbiota-dependent origin. These compounds are partially processed by gut bacteria and can undergo additional hepatic metabolism and urinary excretion, as previously described [5].

Finally, microbiota-specific metabolites such as methanol and ethanol were also modulated by HFD. These compounds are generated exclusively through microbial activity, methanol being associated with methane metabolism, and ethanol produced via fermentation of pyruvate [5]. Their altered levels suggest shifts in microbial populations and metabolic pathways involved in fermentation and methanogenesis (Figure 6c). Importantly, these microbial changes are consistent with previous observations of diet-induced shifts in microbial diversity and metabolic capacity [7].

These observed correlations indicate that the long-term consumption of an HFD (21 weeks) induces a marked reorganization of metabolite–microbiota correlation patterns, consistent with a state of metabolic dysregulation. Importantly, several underexplored metabolites reported in this study emerged as central nodes within these altered correlation networks. Many of these metabolites displayed significant associations with shifts in the fecal microbiota, suggesting that diet-driven microbial changes may play a key role in modulating host metabolic pathways. Given that these metabolites can be readily detected in fecal samples, the fecal host–gut metabolome represents a valuable non-invasive proxy for evaluating host–microbiota interactions and their long-term metabolic consequences. Furthermore, the sex-specific differences in correlation structures show that metabolic adaptations to diet may follow distinct trajectories in males and females. Notably, several fecal metabolites exhibited sex-specific patterns under a high-fat diet, with correlations in males generally being stronger than those observed in females. These differences align with the higher susceptibility of male rodents to developing steatosis [7] and provide insight into sex-dependent metabolic adaptations, highlighting potential mechanisms underlying the observed variation in disease progression. Taken together, these results underscore the potential of fecal metabolomics not only for biomarker discovery but also for providing mechanistic insight into the microbiota–host crosstalk driving diet-induced metabolic disease. Table 2 provides a summary of the most probable origin of the metabolites analyzed in this study, the metabolic pathways in which they are involved, and the interpretation of their presence in fecal samples based on previously cited studies in the literature and reference databases (KEGG, HMDB).

While this study offers novel insights into fecal metabolites associated with early MASLD, certain limitations should be acknowledged. First, the findings are based on endpoint data from a preclinical MASLD rat model. The study design does not allow us to establish the directionality of the associations, and we cannot fully determine whether these metabolites act as causal factors or consequences of the dietary intervention. Second, fecal microbiota was analyzed using qPCR rather than sequencing-based approaches. While qPCR provides high specificity, sensitivity, and quantitative accuracy for selected bacterial taxa, it does not capture the full diversity of the microbial community. We selected this method to focus on representative families and genera of interest, acknowledging that broader sequencing techniques could offer a more comprehensive overview. Third, although correlations with microbial taxa were explored, causal or mechanistic relationships could not be established, and functional roles of the identified metabolites remain to be validated in targeted studies. Fourth, given that most previous metabolomic studies in MASLD have concentrated on SCFAs or serum-based metabolites, direct evidence linking the underexplored fecal host–microbiota metabolites identified here to MASLD is limited. Nonetheless, their strong and diet-dependent correlations with the gut microbiota suggest they may represent overlooked metabolic signatures of MASLD progression. Despite these limitations, this study expands our understanding of gut–liver metabolic interplay and highlights the utility of fecal metabolomics in MASLD research.

## 5. Conclusions

In summary, this study investigated a set of underexplored fecal metabolites beyond SCFAs in a validated in vivo MASLD model. The findings revealed alterations in hepatic-related metabolites, energy metabolism intermediates, BCAA derivatives, and microbial fermentation products, reflecting a reorganization of both host and microbial metabolism. Network analyses further highlighted sex-specific signatures and patterns of dysregulation consistent with MASLD pathophysiology. Although further mechanistic validation is needed, these metabolites provide functional context within host–microbiota pathways and represent promising non-invasive candidates for biomarker discovery and translational applications in MASLD.

## Figures and Tables

**Table 1:** Studied metabolites in feces samples at end point.

N.	Compound Name	^1^H-NMR Chemical Shift (ppm), and Multiplicity	HMDB	KEGG
1	**2-hydroxyvalerate**	**0.90 (t)**, 1.3 (m), 1.4 (m), 1.6 (m), 1.7 (m), 4.0 (m)	HMDB0001863	-
2	**2-oxoglutarate**	**2.4 (t)**, 3.0 (t)	HMDB0000208	C00026
3	**2-oxoisocaproate**	**0.9 (d)**, 2.1 (m), 2.6 (d)	HMDB0000695	C00233
4	**3-aminoisobutyrate**	**1.2 (d)**, 2.6 (m), 3.0 (m), 3.1 (m)	HMDB0003911	C01205
5	**3-hydroxyphenylacetate**	3.5 (s), 6.8 (d), 6.8 (s), 6.9 (d), **7.2 (t)**	HMDB0000440	C05593
6	**3-methyl-2-oxovalerate**	0.9 (t), **1.1 (d)**, 1.4 (m), 1.7 (m), 2.9 (m)	HMDB0000491	C00671
7	**3-phenylpropionate**	**2.5 (m)**, 2.9 (m), 7.3 (t), 7.3 (d), 7.4 (t)	HMDB0000764	C05629
8	**Acetoacetate**	**2.3 (s)**, 3.4 (s)	HMDB0000060	C00164
9	**Cholate**	**0.7 (s)**, 0.9 (s), 1.0 (d, td), 1.2 (m), 1.3 (m), 1.4 (m), 1.5 (d), 1.6 (m), 1.7 (m), 1.8 (m), 1.9 (m), 2.0 (d, m), 2.1 (m), 2.2 (m), 3.5 (m), 3.9 (d), 4.1 (s)	HMDB0000619	C00695
10	**Ethanol**	**1.2 (t)**, 3.6 (q)	HMDB0000108	C00469
11	(L)-**Fucose**	**1.2 (d)**, 3.4 (q), 3.6 (dd), 3.7 (d), 3.8 (d, dd, q), 3.9 (dd), 4.0 (t, m), 4.1 (q), 4.2 (q), 4.5 (d), 5.2 (d), 5.3 (d)	HMDB0000174	C01019
12	**Glycocholate**	**0.7 (s)**, 0.9 (s), 1.0 (d, m), 1.1 (m), 1.3 (m), 1.4 (m), 1.5 (m), 1.6 (m), 1.7 (m), 1.8 (m), 1.9 (m), 2.0 (q), 2.1 (dt), 2.2 (m), 2.4 (m), 3.5 (m), 3.7 (dd), 3.8 (dd), 3.9 (s), 4.0 (s), 7.9 (t)	HMDB0000138	C01921
13	**Lactate**	**1.3 (d)**, 4.1 (q)	HMDB0000190	C00186
14	**Malonate**	**3.1 (s)**	-	C00383
15	**Methanol**	**3.4 (s)**	HMDB0001875	C00132
16	**L-Methionine**	2.1 (s, m), 2.2 (m), **2.6 (t)**, 3.9 (q)	HMDB0000696	C00073
17	**L-Phenylalanine**	3.1 (q), 3.3 (dd), 4.0 (q), 7.3 (d), **7.4 (t)**	HMDB0000159	C00079
18	**L-Tyrosine**	3.0 (q), 3.2 (dd), 3.9 (q), **6.9 (d)**, 7.2 (d)	HMDB0000158	C00082

The abbreviations (s) singlet, (d) doublet, (dd) double doublet, (t) triplet, (dt) double triplet, (q) quartet, and (m) multiplet refer to the number of peaks observed in the ^1^H-NMR spectrum. The reference peak selected for each analyzed metabolite is highlighted in **bold**.

**Table 2:** Overview of the identified metabolites.

Metabolite	Probable Origin	Metabolic Pathway	Biological Significance in Feces
2-hydroxyvalerate	Host and Microbiota	Branched-chain fatty acid metabolism	From BCAA or intermediate in energy metabolism
2-oxglutarate	Host and Microbiota	TCA cycle	Key intermediate in energy metabolism
2-oxoisocaproate	Host and Microbiota	Leucine metabolism	Intermediate in leucine catabolism
3-aminoisobutyrate	Host and Microbiota	BCAA or thymine metabolism	Microbial metabolism changes
3-hydroxyphenylacetate	Microbiota	Phenylalanine metabolism	Indicator of changes in microbial diversity
3-methyl-2-isovalerate	Host and Microbiota	Isoleucine and propanoyl-CoA metabolism	Microbial metabolism changes
3-Phenylpropionate	Microbiota	Phenylalanine metabolism	Indicator of changes in microbial diversity
Acetoacetate	Host and Microbiota	Acetyl-CoA metabolism	Elevated in increased lipid oxidation or ketogenic state
Cholate	Host	Primary BA metabolism	Marker of liver function or indicator of changes in enterohepatic activity
Ethanol	Microbiota	Pyruvate fermentation	Indicator of changes in microbial diversity
(L)-Fucose	Host	Carbohydrate metabolism (fructose and mannose)	Liver metabolism imbalance
Glycocholate	Host and Microbiota	BA metabolism; conjugation/deconjugation processes	Marker of liver function or indicator of changes in enterohepatic activity
Lactate	Host and Microbiota	Anaerobic glycolysis	Indicator of changes in microbial diversity
Malonate	Host and Microbiota	TCA cycle inhibitor/FA metabolism	Regulator of energy metabolism
Methanol	Microbiota	Methane metabolism	Indicator of changes in microbial diversity
Methionine	Host and Microbiota	Amino acid metabolism	Involved in methylation and antioxidant defense
Phenylalanine	Host and Microbiota	Aromatic amino acid metabolism	Precursor of microbial products
Tyrosine	Host and Microbiota	Aromatic amino acid metabolism	Precursor of microbial products; also it can be a neurotransmitter precursor

Table 2 is based on previously cited studies in the literature and reference databases (KEGG, HMDB).

## Data Availability

The microbiota data employed for the correlations were reported in a previous article by the same first author, Martin-Grau, M. (2025) [7] (DOI: 10.3390/ijms26031288). Likewise, Section 2 refers to the model described in the same article, where the full information can be found. Finally, the data presented in this study are available on request from the corresponding authors.

## Supplementary Materials

The following supporting information can be downloaded at https://www.mdpi.com/article/10.3390/metabo15100660/s1. We have included the metabolomics data as well as the microbiota data used to perform all the analyses.

## Abbreviations

The following abbreviations are used in this manuscript:

BA	Bile acid
BCAA	Branched amino acids
CTL	Chow diet
HFD	High-fat diet
HMDB	Human Metabolome Database
KEGG	Kyoto Encyclopedia of Genes and Genomes
MASLD	Metabolic dysfunction-associated steatotic liver disease
NMR	Nuclear magnetic resonance
PCA	Principal Component Analysis
SCFA	Short-chain fatty acids
TMAO	Trimethylamine N-oxide
